# Supplementary material for: Host-Associated Distribution of Two Novel Mammarenaviruses in Rodents from Southern Africa
Source: Viruses. 2022 Dec 29;15(1):99. doi: 10.3390/v15010099 (PMC9861163; doi:10.3390/v15010099)
Supplement: Supplementary file 1 [file viruses-15-00099-s001.zip › viruses-2118212-supplementary.pdf]

Supplementary materials

Host-associated distribution of two novel mammarenaviruses in rodents from Southern Africa

Marike Geldenhuys [1], Jacqueline Weyer [1,2,3], Teresa Kearney [4,5], and Wanda Markotter [1*]

Supplementary figures

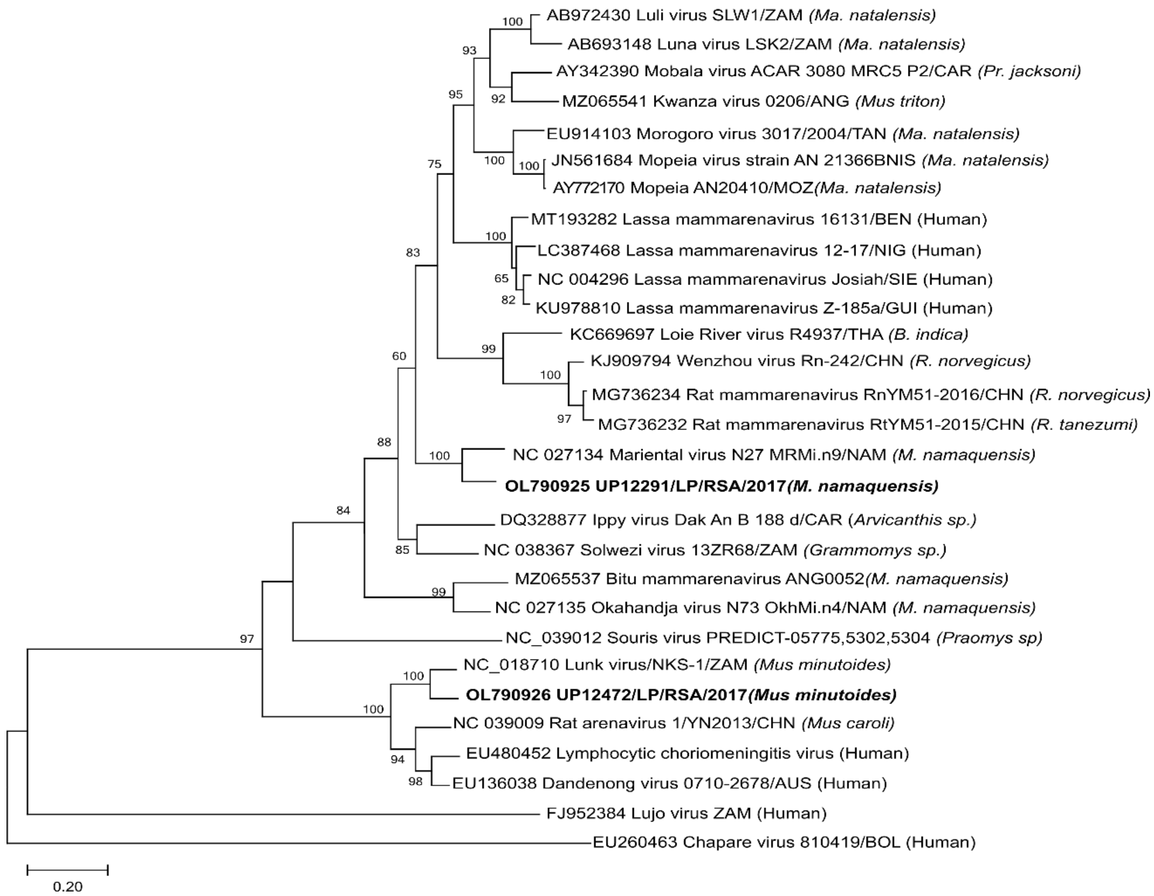

Figure S1: Phylogeny of a 514 amino acid region of the S segment glycoprotein gene constructed by maximum likelihood approach using the LG model with gamma distribution and 1000 bootstrap replicates in MegaX. Sequences in bold denote those reported from this study. All host species of origin are indicated, including countries from which viruses were reported as standard 3-letter country codes.

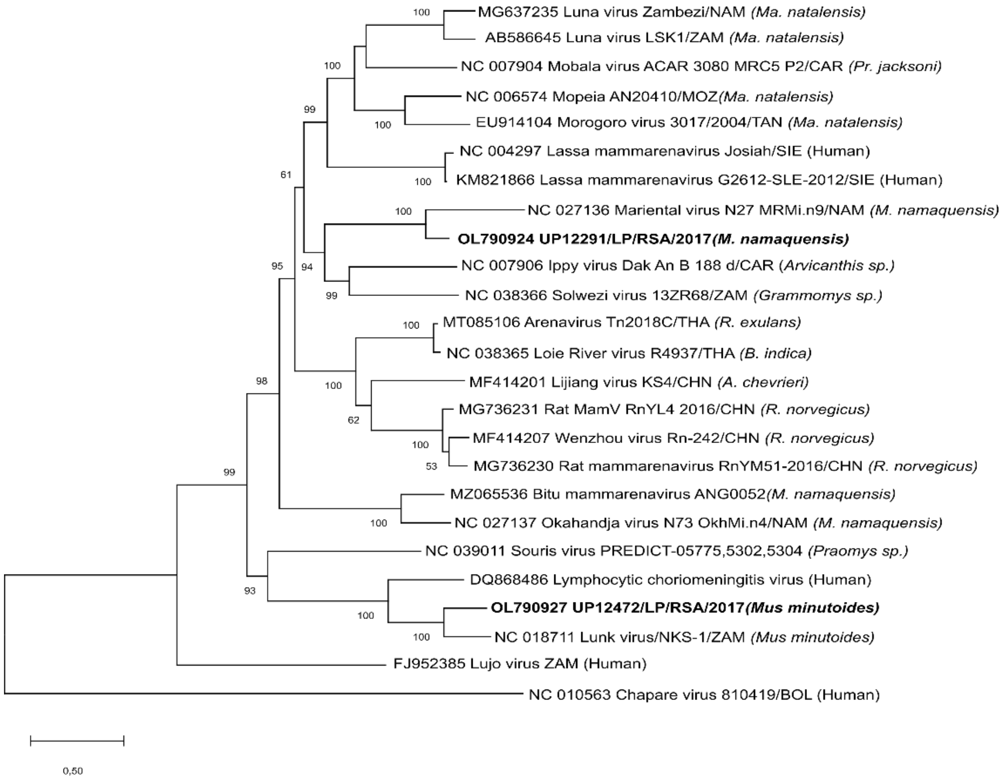

Figure S2: Phylogeny of the arenavirus L gene (approximately 6906 nucleotides) constructed by maximum likelihood approach using the general time reversible (GTR) model with gamma distribution and invariant sites and 1000 bootstrap replicates in MegaX. Sequences in bold denote those reported from this study. Host species of origin are indicated and countries from which viruses were reported are indicated in sequence names as standard 3-letter country codes.

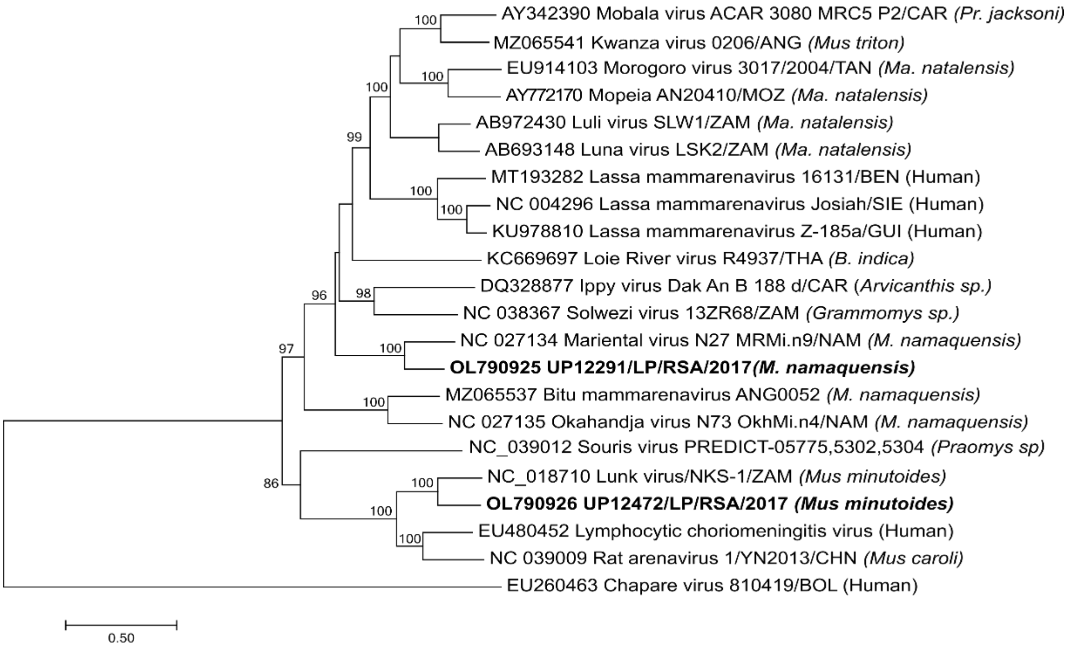

Figure S3: Phylogeny of the S segment (approximately 3134 nucleotides) constructed by maximum likelihood approach using the general time reversible (GTR) model with gamma distribution and invariant sites and 1000 bootstrap replicates in MegaX. Sequences in bold denote those reported from this study. Host species of origin are indicated and countries from which viruses were reported are indicated in sequence names as standard 3-letter country codes.

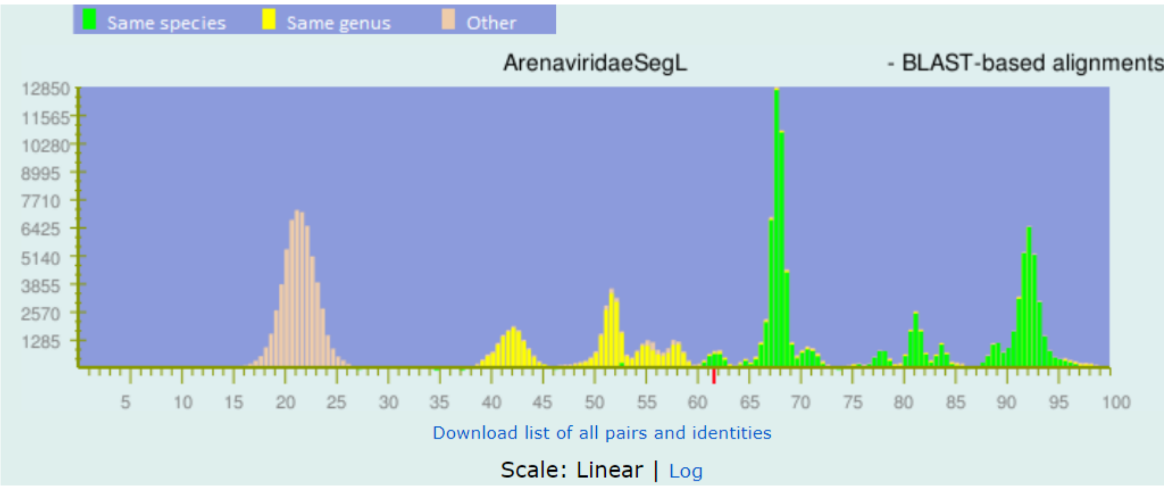

Figure S4: PAirwise Sequence Comparison (PASC) program output of UP12291/M. namaquensis/LP/RSA/2017 L segmernt from NCBI, with closest relative Mariental virus N27 MRMi.n9.

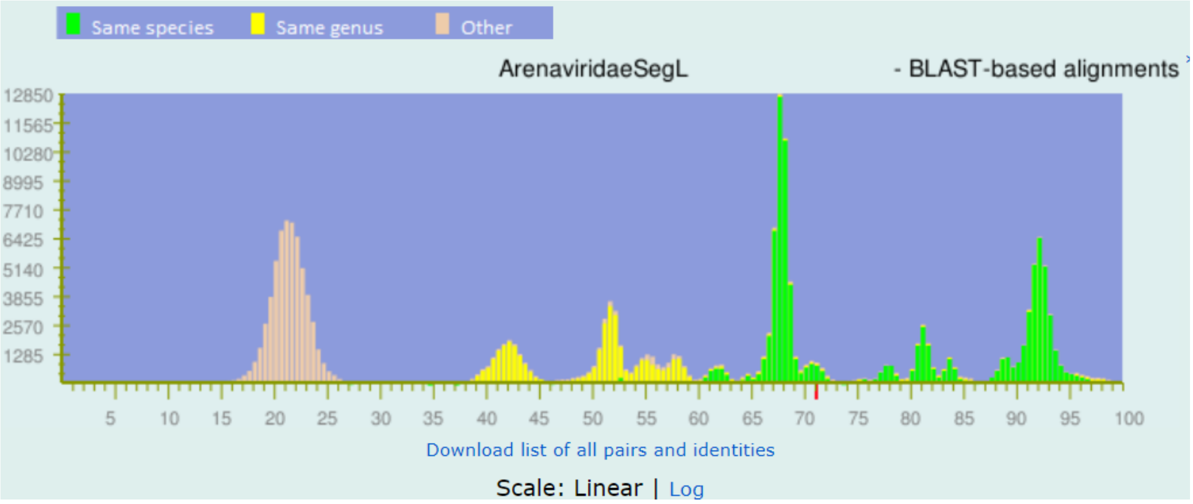

Figure S5: PAirwise Sequence Comparison (PASC) program output of UP12472/M. minutoides/LP/RSA/2017 L segmernt from NCBI, with closest relative Lunk mammarenavirus.

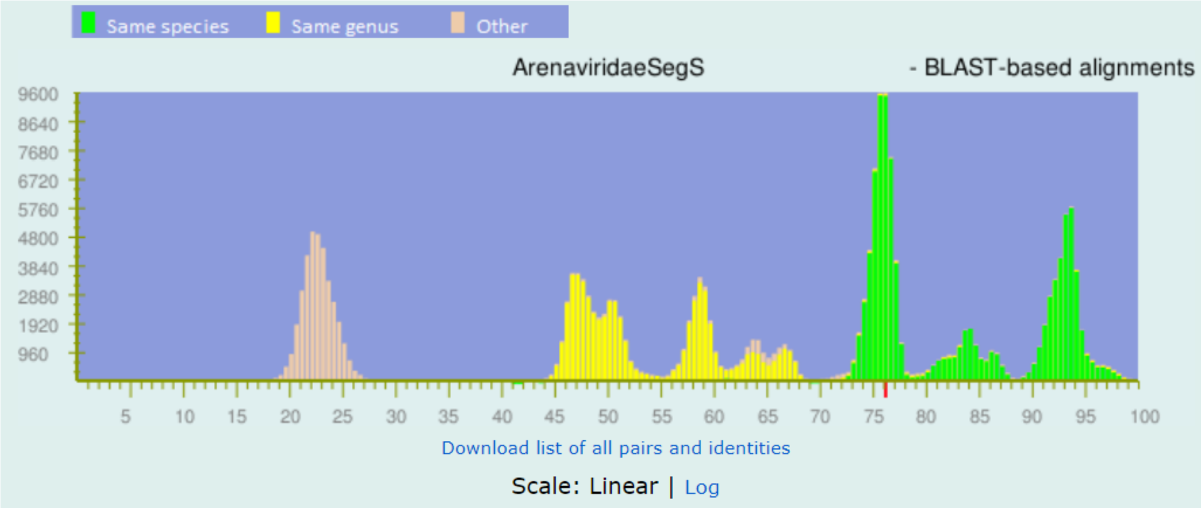

Figure S6: PAirwise Sequence Comparison (PASC) program output of UP12291/M. namaquensis/LP/RSA/2017 S segmernt from NCBI, with closest relative Mariental virus N27 MRMi.n9.

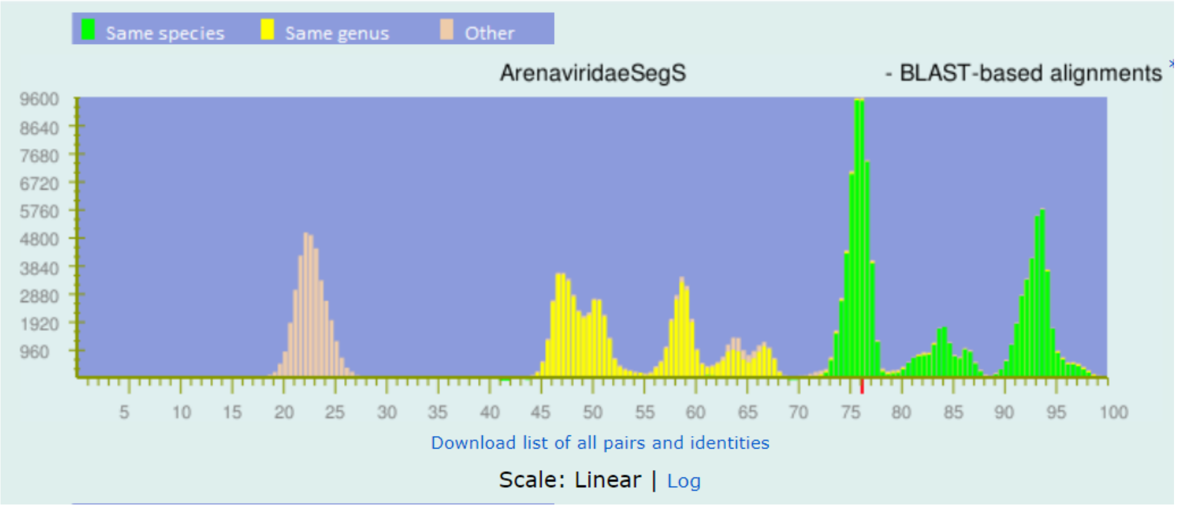

Figure S7: PAirwise Sequence Comparison (PASC) program output of UP12472/M. minutoides/LP/RSA/2017 S segmernt from NCBI, with closest relative Lunk mammarenavirus.

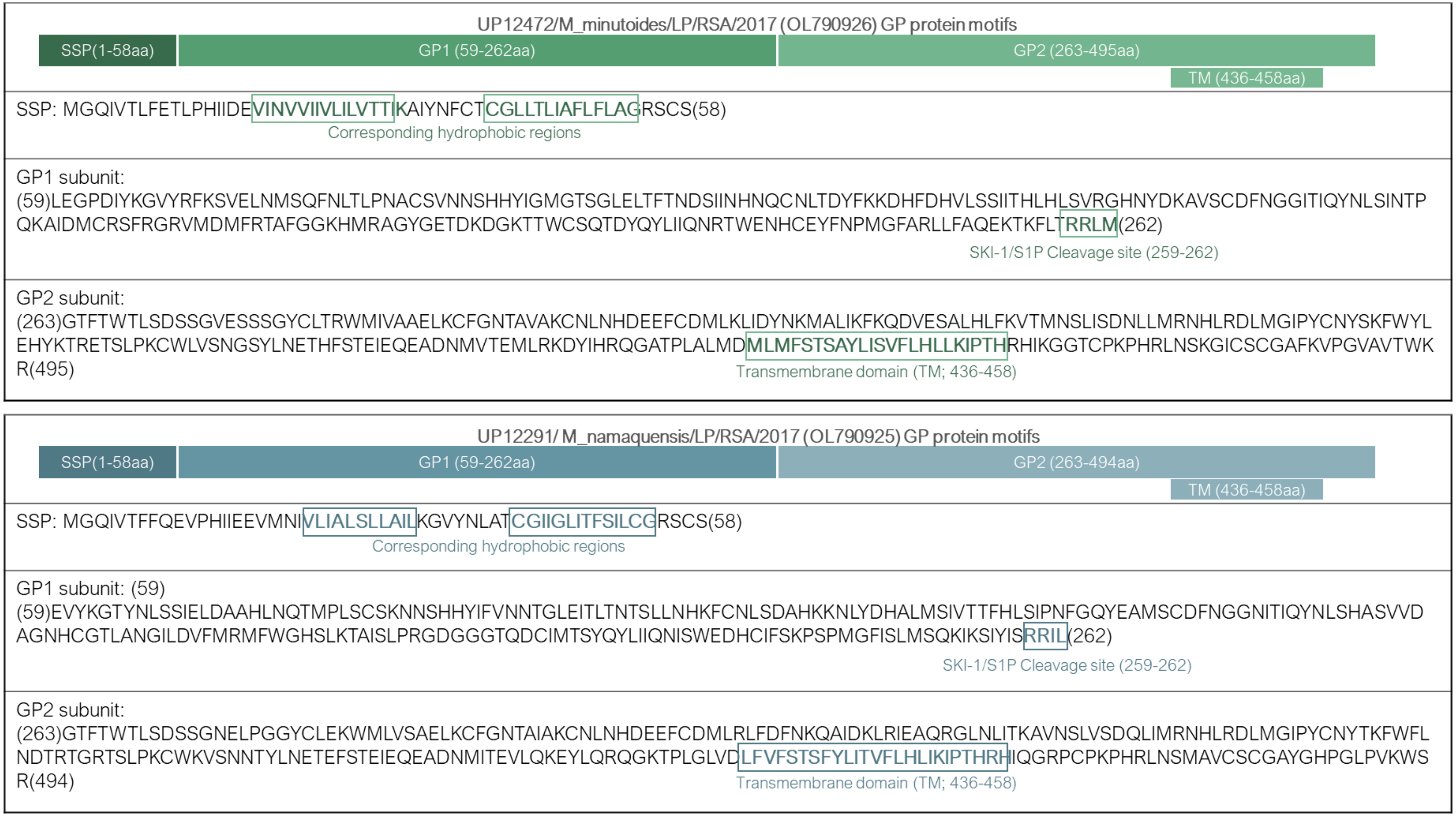

Figure S8: Schematic indicating the annotations of the GPC protein. The signal peptide (SSP), G1 and G2 regions are indicted with hydrophobic regions in SSP, SKI-1/S1P cleavage sites and transmembrane domain highlighted. Top half of the schematic shows the annotations for UP12472/M_minutoides/LP/RSA/2017 (OL790926) GP protein motifs and the bottom for UP12291/ M_namaquensis/LP/RSA/2017 (OL790925) GP protein motifs.

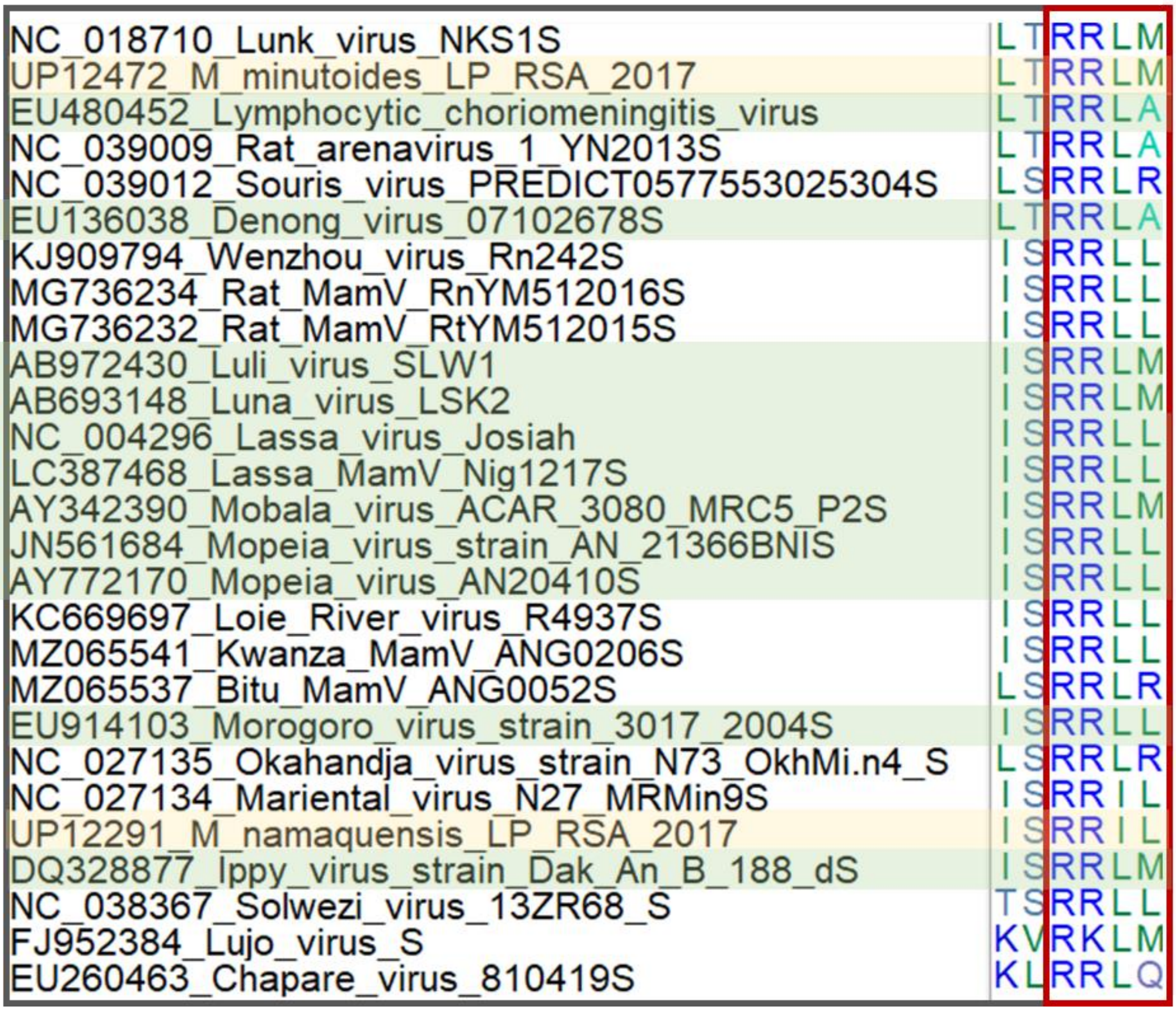

Figure S9: Alignment of the RRLL recognition, SP1/SKI-1 cleavage site of mammarenaviruses. The terminal 6 amino acid residues of the GP1 submit are depicted with the recognition site boxed in red. The alignment contains mainly Old-World arenaviruses, as they represent alpha-dystroglycan receptor users, with the viruses highlighted in green having been confirmed as users of alpha-dystroglycan. Viruses highlighted in yellow represent those described here.

Table S1 Surveillance data:

Laboratory number	Field number	Museum number	Date collected	Species ID (Morphological)	Province	Sex	Samples	Arenavirus Results	Hantavirus Results
UP 4961	UP 4961	TM49121	27-Feb-15	*Aethomys ineptus s.l.*	Limpopo	F	Fe	Neg	Neg
UP 4962	UP 4962	TM49197	27-Feb-15	*Gerbilliscus leucogaster*	Limpopo	M	Fe	Neg	Neg
UP 4963	UP 4963	TM49122	27-Feb-15	*Aethomys ineptus s.l.*	Limpopo	M	Fe	Neg	Neg
UP 4967	UP 4967	TM49137	27-Feb-15	*Aethomys ineptus s.l.*	Limpopo	F	Fe	Neg	Neg
UP 4968	UP 4968	TM49210	27-Feb-15	*Saccostomus campestris*	Limpopo	F	Fe	Neg	Neg

UP 4969	UP 4969	TM49123	27-Feb-15	*Aethomys ineptus s.l.*	Limpopo	M	Fe	Neg	Neg
UP 4973	UP 4973	TM49216	27-Feb-15	*Graphiurus murinus*	Limpopo	M	Fe	Neg	Neg
UP 4995	UP 4995	TM49199	01-Mar-15	*Mastomys natalensis s.l.*	Limpopo	M	Fe	Neg	Neg
UP 5011	UP 5011	TM49140	01-Mar-15	*Steatomys sp.*	Limpopo	M	Fe	Neg	Neg
UP 5012	UP 5012	TM49124	01-Mar-15	*Aethomys ineptus s.l.*	Limpopo	M	Fe	Neg	Neg
UP 5015	UP 5015	TM49141	01-Mar-15	*Steatomys sp.*	Limpopo	F	Fe	Neg	Neg
UP 5353	UPR 003	TM49208	12-May-15	*Elephantulus brachyrhynchus*	Limpopo	M	Fe	Neg	Neg
UP 5354	UPR 004	TM49200	12-May-15	*Mastomys natalensis s.l.*	Limpopo	M	Fe	Neg	Neg
UP 5355	UPR 005	TM49209	12-May-15	*Elephantulus brachyrhynchus*	Limpopo	M	Fe	Neg	Neg
UP 5356	UPR 006	TM49201	12-May-15	*Mastomys natalensis s.l.*	Limpopo	M	Fe	Neg	Neg
UP 5525	UPR 086	TM49126	12-July-15	*Aethomys ineptus s.l.*	Limpopo	M	Fe	Neg	Neg
UP 5934	UPR 091	TM49220	15-Sep-15	*Mus (Nannomys) sp.*	Limpopo	F	Fe	Neg	Neg
UP 5935	UPR 092	TM49215	15-Sep-15	*Micaelamys namaquensis*	Limpopo	M	Fe	Neg	Neg
UP 5939	UPR 096	TM49221	15-Sep-15	*Mus (Nannomys) sp.*	Limpopo	M	Fe	Neg	Neg
UP 12001	UPR 141	TM49202	13-Nov-15	*Mastomys natalensis s.l.*	Limpopo	M	Ki	Neg	Neg
UP 12002	UPR 142	TM49138	13-Nov-15	*Aethomys ineptus s.l.*	Limpopo	F	Ki	Neg	Neg
UP 12003	UPR 143	TM49131	13-Nov-15	*Aethomys ineptus s.l.*	Limpopo	F	Ki	Neg	Neg
UP 12004	UPR 144	TM49143	13-Nov-15	*Steatomys sp.*	Limpopo	F	Ki	Neg	Neg
UP 12005	UPR 145	TM49129	13-Nov-15	*Aethomys ineptus s.l.*	Limpopo	F	Ki	Neg	Neg
UP 12006	UPR 146	TM49130	13-Nov-15	*Aethomys ineptus s.l.*	Limpopo	F	Ki	Neg	Neg
UP 12007	UPR 147	TM49144	13-Nov-15	*Steatomys sp.*	Limpopo	M	Ki	Neg	Neg
UP 12008	UPR 148	TM49222	13-Nov-15	*Mus (Nannomys) sp.*	Limpopo	M	Ki	Neg	Neg
UP 12009	UPR 149	TM49132	13-Nov-15	*Aethomys ineptus s.l.*	Limpopo	F	Ki	Neg	Neg
UP 12010	UPR 150	TM49203	19-Jan-16	*Mastomys natalensis s.l.*	Limpopo	F	Fe /Ki	Neg	Neg
UP 12011	UPR 151	TM49145	19-Jan-16	*Steatomys sp.*	Limpopo	F	Fe/ Ki	Neg	Neg
UP 12012	UPR 152	TM49198	19-Jan-16	*Gerbilliscus leucogaster*	Limpopo	F	Fe/ Ki	Neg	Neg
UP 12013	UPR 153	TM49204	19-Jan-16	*Mastomys natalensis s.l.*	Limpopo	F	Ki	Neg	Neg
UP 12014	UPR 154	TM49205	19-Jan-16	*Mastomys natalensis s.l.*	Limpopo	F	Ki	Neg	Neg
UP 12016	UPR 156	TM49139	20-Jan-16	*Aethomys ineptus s.l.*	Limpopo	M	Ki	Neg	Neg
UP 12017	UPR 157	TM49133	20-Jan-16	*Aethomys ineptus s.l.*	Limpopo	M	Fe/ Ki	Neg	Neg
UP 12018	UPR 158	TM49206	20-Jan-16	*Mastomys natalensis s.l.*	Limpopo	M	Ki	Neg	Neg
UP 12019	UPR 159	TM49211	20-Jan-16	*Saccostomus campestris*	Limpopo	M	Ki	Neg	Neg
UP 12020	UPR 160	TM49207	20-Jan-16	*Mastomys natalensis s.l.*	Limpopo	F	Ki	Neg	Neg
UP 12021	UPR 161	TM49134	20-Jan-16	*Aethomys ineptus s.l.*	Limpopo	M	Ki	Neg	Neg
UP 12022	UPR 162	TM49135	20-Jan-16	*Aethomys ineptus s.l.*	Limpopo	F	Ki	Neg	Neg
UP 12023	UPR 163	TM49212	21-Jan-16	*Saccostomus campestris*	Limpopo	F	Fe/ Ki	Neg	Neg
UP 12024	UPR 164	TM49136	21-Jan-16	*Aethomys ineptus s.l.*	Limpopo	F	Fe/ Ki	Neg	Neg
UP 12061	UPR 201	TM49242	05-Apr-16	*Aethomys ineptus s.l.*	Limpopo	F	Ki	Neg	Neg
UP 12062	UPR 202	Released; no voucher	05-Apr-16	*Saccostomus campestris*	Limpopo	M	Fe	Neg	Neg
UP 12065	UPR 203	TM49243	06-Apr-16	*Saccostomus campestris*	Limpopo	M	Fe/ Ki	Neg	Neg
UP 12066	UPR 204	TM49224	06-Apr-16	*Graphiurus murinus*	Limpopo	F	Fe/ Ki	Neg	Neg
UP 12067	UPR 205	TM49225	06-Apr-16	*Aethomys ineptus s.l.*	Limpopo	F	Fe/ Ki	Neg	Neg
UP 12068	UPR 206	Released; no voucher	07-Apr-16	*Saccostomus campestris*	Limpopo	F	Fe	Neg	Neg

UP 12070	UPR 207	TM49226	07-Apr-16	*Aethomys ineptus s.l.*	Limpopo	F	Ki	Neg	Neg
UP 12071	UPR 208	TM49227	08-Apr-16	*Aethomys ineptus s.l.*	Limpopo	F	Fe/ Ki	Neg	Neg
UP 12072	UPR 209	TM49244	08-Apr-16	*Gerbilliscus leucogaster*	Limpopo	F	Ki	Neg	Neg
UP 12073	UPR 210	TM49228	08-Apr-16	*Aethomys ineptus s.l.*	Limpopo	M	Fe/ Ki	Neg	Neg
UP 12074	UPR 211	TM49229	08-Apr-16	*Aethomys ineptus s.l.*	Limpopo	M	Ki	Neg	Neg
UP 12075	UPR 212	Released; no voucher	07-Jun-16	*Mastomys natalensis s.l.*	Limpopo	M	Fe	Neg	Neg
UP 12077	UPR 213	Released; no voucher	07-Jun-16	*Saccostomus campestris*	Limpopo	F	Fe	Neg	Neg
UP 12081	UPR 214	TM49230	07-Jun-16	*Aethomys ineptus s.l.*	Limpopo	M	Fe/ Ki	Neg	Neg
UP 12082	UPR 215	TM49245	07-Jun-16	*Aethomys ineptus s.l.*	Limpopo	F	Fe/ Ki	Neg	Neg
UP 12083	UPR 216	TM49246	07-Jun-16	*Acomys selousi*	Limpopo	F	Fe/ Ki	Neg	Neg
UP 12084	UPR 217	TM49247	08-Jun-16	*Saccostomus campestris*	Limpopo	M	Fe/ Ki	Neg	Neg
UP 12085	UPR 218	TM49231	08-Jun-16	*Micaelamys namaquensis*	Limpopo	M	Fe/ Ki	Neg	Neg
UP 12086	UPR 219	TM49248	08-Jun-16	*Gerbilliscus leucogaster*	Limpopo	M	Fe/ Ki	Neg	Neg
UP 12087	UPR 220	TM49249	08-Jun-16	*Aethomys ineptus s.l.*	Limpopo	M	Fe/ Ki	Neg	Neg
UP 12088	UPR 221	TM49232	09-Jun-16	*Aethomys ineptus s.l.*	Limpopo	F	Fe/ Ki	Neg	Neg
UP 12089	UPR 222	Released; no voucher	09-Jun-16	*Mastomys natalensis s.l.*	Limpopo	M	Fe	Neg	Neg
UP 12090	UPR 223	Released; no voucher	10-Jun-16	*Steatomys sp.*	Limpopo	M	Fe	Neg	Neg
UP 12132	UPR 265	TM49250	06-Sep-16	*Aethomys ineptus s.l.*	Limpopo	F	Fe/ Ki	Neg	Neg
UP 12133	UPR 266	TM49251	06-Sep-16	*Gerbilliscus leucogaster*	Limpopo	M	Fe/ Ki	Neg	Neg
UP 12134	UPR 267	Released; no voucher	06-Sep-16	*Micaelamys namaquensis*	Limpopo	F	Fe	Neg	Neg
UP 12137	UPR 268	TM49252	06-Sep-16	*Micaelamys namaquensis*	Limpopo	M	Fe/ Ki	Neg	Neg
UP 12138	UPR 269	TM49253	06-Sep-16	*Micaelamys namaquensis*	Limpopo	M	Ki	Neg	Neg
UP 12139	UPR 270	TM50720	07-Sep-16	*Gerbilliscus leucogaster*	Limpopo	M	Ki	Neg	Neg
UP 12140	UPR 271	Released; no voucher	07-Sep-16	*Micaelamys namaquensis*	Limpopo	M	Fe	Neg	Neg
UP 12142	UPR 272	TM49233	07-Sep-16	*Elephantulus brachyrhynchus*	Limpopo	M	Fe/ Ki	Neg	Neg
UP 12143	UPR 273	Released; no voucher	08-Sep-16	*Micaelamys namaquensis*	Limpopo	M	Fe	Neg	Neg
UP 12144	UPR 274	TM50723	08-Sep-16	*Micaelamys namaquensis*	Limpopo	F	Ki	Neg	Neg
UP 12145	UPR 275	TM49234	09-Sep-16	*Micaelamys namaquensis*	Limpopo	F	Fe/ Ki	Neg	Neg
UP 12146	UPR 276	TM49235	09-Sep-16	*Acomys selousi*	Limpopo	M	Fe/ Ki	Neg	Neg
UP 12147	UPR 277	TM49254	09-Sep-16	*Steatomys sp.*	Limpopo	F	Fe/ Ki	Neg	Neg
UP 12148	UPR 278	TM49255	09-Sep-16	*Saccostomus campestris*	Limpopo	M	Fe/ Ki	Neg	Neg
UP 12149	UPR 279	Released; no voucher	09-Sep-16	*Saccostomus campestris*	Limpopo	M	Fe	Neg	Neg
UP 12151	UPR 280	TM50724	09-Sep-16	*Saccostomus campestris*	Limpopo	M	Ki	Neg	Neg
UP 12152	UPR 281	Released; no voucher-	10-Nov-16	*Acomys selousi*	Limpopo	M	Fe	Neg	Neg
UP 12154	UPR 282	TM49256	08-Nov-16	*Micaelamys namaquensis*	Limpopo	M	Ki	Neg	Neg
UP 12155	UPR 283	Released; no voucher	08-Nov-16	*Mastomys natalensis s.l.*	Limpopo	F	Fe	Neg	Neg
UP 12157	UPR 284	TM49257	08-Nov-16	*Aethomys ineptus s.l.*	Limpopo	F	Fe/ Ki	Neg	Neg
UP 12158	UPR 285	Released; no voucher -	08-Nov-16	*Saccostomus campestris*	Limpopo	M	Fe/ Ki	Neg	Neg
UP 12163	UPR 286	Released; no voucher -	08-Nov-16	*Gerbilliscus leucogaster*	Limpopo	F	Fe	Neg	Neg

UP 12165	UPR 287	TM49258	08-Nov-16	*Aethomys ineptus s.l.*	Limpopo	F	Fe/ Ki	Neg	Neg
UP 12166	UPR 288	TM49259	09-Nov-16	*Gerbilliscus leucogaster*	Limpopo	M	Ki	Neg	Neg
UP 12167	UPR 289	TM49260	09-Nov-16	*Aethomys ineptus s.l.*	Limpopo	F	Fe/ Ki	Neg	Neg
UP 12168	UPR 290	TM49261	10-Nov-16	*Micaelamys namaquensis*	Limpopo	M	Fe/ Ki	Neg	Neg
UP 12169	UPR 291	TM49262	10-Nov-16	*Mus (Nannomys) minutoides s.l.*	Limpopo	F	Fe/ Ki	Neg	Neg
UP 12170	UPR 292	TM49263	10-Nov-16	*Aethomys ineptus s.l.*	Limpopo	M	Fe/ Ki	Neg	Neg
UP 12172	UPR 294	TM49264	11-Nov-16	*Mus (Nannomys) minutoides s.l.*	Limpopo	M	Ki	Neg	Neg
UP 12173	UPR 295	TM49265	11-Nov-16	*Aethomys ineptus s.l.*	Limpopo	F	Ki	Neg	Neg
UP 12176	UPR 298	TM49266	07-Feb-17	*Saccostomus campestris*	Limpopo	M	Ki	Neg	Neg
UP 12177	UPR 299	TM49267	08-Feb-17	*Aethomys ineptus s.l.*	Limpopo	M	Fe/ Ki	Neg	Neg
UP 12178	UPR 300	TM49268	08-Feb-17	*Aethomys ineptus s.l.*	Limpopo	M	Fe/ Ki	Neg	Neg
UP 12179	UPR 301	TM49269	08-Feb-17	*Aethomys ineptus s.l.*	Limpopo	F	Fe/ Ki	Neg	Neg
UP 12180	UPR 302	TM49270	09-Feb-17	*Aethomys ineptus s.l.*	Limpopo	M	Fe/ Ki	Neg	Neg
UP 12181	UPR 303	TM49271	09-Feb-17	*Aethomys ineptus s.l.*	Limpopo	F	Fe/ Ki	Neg	Neg
UP 12182	UPR 304	TM49272	10-Feb-17	*Saccostomus campestris*	Limpopo	F	Ki	Neg	Neg
UP 12183	UPR 305	Released; no voucher -	27-Mar-17	*Gerbilliscus leucogaster*	Limpopo	F	Fe	Neg	Neg
UP 12184	UPR 306	TM50725	27-Mar-17	*Gerbilliscus leucogaster*	Limpopo	F	Fe/ Ki	Neg	Neg
UP 12185	UPR 307	TM50540	27-Mar-17	*Gerbilliscus leucogaster*	Limpopo	F	Fe/ Ki	Neg	Neg
UP 12186	UPR 308	Released; no voucher -	27-Mar-17	*Gerbilliscus leucogaster*	Limpopo	F	Fe	Neg	Neg
UP 12187	UPR 309	TM50541	27-Mar-17	*Gerbilliscus leucogaster*	Limpopo	F	Ki	Neg	Neg
UP 12188	UPR 310	TM50726	27-Mar-17	*Saccostomus campestris*	Limpopo	M	Fe/ Ki	Neg	Neg
UP 12189	UPR 311	TM50727	27-Mar-17	*Saccostomus campestris*	Limpopo	F	Fe/ Ki	Neg	Neg
UP 12190	UPR 312	TM50728	27-Mar-17	*Steatomys sp.*	Limpopo	M	Fe/ Ki	Neg	Neg
UP 12191	UPR 313	TM50729	27-Mar-17	*Steatomys sp.*	Limpopo	F	Fe/ Ki	Neg	Neg
UP 12192	UPR 314	TM50730	28-Mar-17	*Steatomys sp.*	Limpopo	M	Fe/ Ki	Neg	Neg
UP 12193	UPR 315	TM50542	28-Mar-17	*Gerbilliscus leucogaster*	Limpopo	M	Fe/ Ki	Neg	Neg
UP 12194	UPR 316	NHCPHE_MAM-20	28-Mar-17	*Gerbilliscus leucogaster*	Limpopo	F	Fe/ Ki	Neg	Neg
UP 12195	UPR 317	NHCPHE_MAM-21	28-Mar-17	*Gerbilliscus leucogaster*	Limpopo	M	Fe/ Ki	Neg	Neg
UP 12197	UPR 319	NHCPHE_MAM-22	28-Mar-17	*Gerbilliscus leucogaster*	Limpopo	M	Fe/ Ki	Neg	Neg
UP 12198	UPR 320	Released; no voucher -	28-Mar-17	*Gerbilliscus leucogaster*	Limpopo	F	Fe	Neg	Neg
UP 12199	UPR 321	Released; no voucher -	28-Mar-17	*Gerbilliscus leucogaster*	Limpopo	F	Fe	Neg	Neg
UP 12200	UPR 322	Released; no voucher -	28-Mar-17	*Gerbilliscus leucogaster*	Limpopo	M	Fe	Neg	Neg
UP 12201	UPR 323	TM50731	28-Mar-17	*Steatomys sp.*	Limpopo	M	Fe/ Ki	Neg	Neg
UP 12202	UPR 324	NHCPHE_MAM-23	28-Mar-17	*Gerbilliscus leucogaster*	Limpopo	F	Ki	Neg	Neg
UP 12203	UPR 325	TM50732	28-Mar-17	*Gerbilliscus leucogaster*	Limpopo	M	Fe/ Ki	Neg	Neg
UP 12204	UPR 326	TM50717	28-Mar-17	*Aethomys ineptus s.l.*	Limpopo	F	Fe/ Ki	Neg	Neg
UP 12205	UPR 327	TM50733	28-Mar-17	*Mastomys natalensis s.l.*	Limpopo	M	Fe/ Ki	Neg	Neg
UP 12206	UPR 328	TM50734	28-Mar-17	*Mastomys natalensis s.l.*	Limpopo	F	Fe/ Ki	Neg	Neg
UP 12207	UPR 329	TM50543	28-Mar-17	*Gerbilliscus leucogaster*	Limpopo	M	Fe/ Ki	Neg	Neg

UP 12208	UPR 330	NHCPHE_MAM-3	29-Mar-17	*Gerbilliscus leucogaster*	Limpopo	F	Fe/ Ki	Neg	Neg
UP 12209	UPR 331	TM50735	29-Mar-17	*Mastomys natalensis s.l.*	Limpopo	M	Fe/ Ki	Neg	Neg
UP 12210	UPR 332	TM50736	29-Mar-17	*Mastomys natalensis s.l.*	Limpopo	F	Fe/ Ki	Neg	Neg
UP 12211	UPR 333	TM50737	29-Mar-17	*Steatomys sp.*	Limpopo	F	Fe/ Ki	Neg	Neg
UP 12212	UPR 334	TM50738	29-Mar-17	*Mastomys natalensis s.l.*	Limpopo	F	Fe/ Ki	Neg	Neg
UP 12213	UPR 335	TM49273	29-Mar-17	*Crocidura hirta*	Limpopo	M	Fe/ Ki	Neg	Neg
UP 12214	UPR 336	Released; no voucher	29-Mar-17	*Steatomys sp.*	Limpopo	F	Fe	Neg	Neg
UP 12215	UPR 337	TM50739	29-Mar-17	*Mus (Nannomys) sp.*	Limpopo	F	Fe/ Ki	Neg	Neg
UP 12216	UPR 338	TM50740	29-Mar-17	*Steatomys sp.*	Limpopo	F	Fe/ Ki	Neg	Neg
UP 12217	UPR 339	TM50741	29-Mar-17	*Mastomys natalensis s.l.*	Limpopo	M	Fe/ Ki	Neg	Neg
UP 12218	UPR 340	TM50742	29-Mar-17	*Saccostomus campestris*	Limpopo	M	Fe/ Ki	Neg	Neg
UP 12219	UPR 341	TM50743	29-Mar-17	*Steatomys sp.*	Limpopo	F	Fe/ Ki	Neg	Neg
UP 12220	UPR 342	Released; no voucher	29-Mar-17	*Steatomys sp.*	Limpopo	M	Fe	Neg	Neg
UP 12221	UPR 343	Released; no voucher	29-Mar-17	*Gerbilliscus leucogaster*	Limpopo	F	Fe	Neg	Neg
UP 12222	UPR 344	Released; no voucher	29-Mar-17	*Steatomys sp.*	Limpopo	M	Fe	Neg	Neg
UP 12223	UPR 345	TM50544	29-Mar-17	*Gerbilliscus leucogaster*	Limpopo	M	Fe/ Ki	Neg	Neg
UP 12224	UPR 346	Released; no voucher	29-Mar-17	*Gerbilliscus leucogaster*	Limpopo	F	Fe	Neg	Neg
UP 12225	UPR 347	Released; no voucher	29-Mar-17	*Mastomys natalensis s.l.*	Limpopo	F	Fe	Neg	Neg
UP 12226	UPR 348	Released; no voucher	29-Mar-17	*Gerbilliscus leucogaster*	Limpopo	M	Fe	Neg	Neg
UP 12227	UPR 349	Released; no voucher	29-Mar-17	*Gerbilliscus leucogaster*	Limpopo	F	Fe	Neg	Neg
UP 12228	UPR 350	Released; no voucher	29-Mar-17	*Gerbilliscus leucogaster*	Limpopo	M	Fe	Neg	Neg
UP 12229	UPR 351	Released; no voucher	29-Mar-17	*Steatomys sp.*	Limpopo	F	Fe	Neg	Neg
UP 12230	UPR 352	Released; no voucher	29-Mar-17	*Steatomys sp.*	Limpopo	M	Fe	Neg	Neg
UP 12231	UPR 353	Released; no voucher	29-Mar-17	*Steatomys sp.*	Limpopo	M	Fe	Neg	Neg
UP 12232	UPR 354	Released; no voucher	29-Mar-17	*Steatomys sp.*	Limpopo	M	Fe	Neg	Neg
UP 12233	UPR 355	Released; no voucher	29-Mar-17	*Gerbilliscus leucogaster*	Limpopo	M	Fe	Neg	Neg
UP 12234	UPR 356	TM50744	29-Mar-17	*Gerbilliscus leucogaster*	Limpopo	M	Ki	Neg	Neg
UP 12235	UPR 357	Released; no voucher	29-Mar-17	*Gerbilliscus leucogaster*	Limpopo	M	Fe	Neg	Neg
UP 12236	UPR 358	TM50745	30-Mar-17	*Saccostomus campestris*	Limpopo	M	Fe/ Ki	Neg	Neg
UP 12237	UPR 359	TM50746	30-Mar-17	*Mus (Nannomys) sp.*	Limpopo	M	Ki	Neg	Neg
UP 12238	UPR 360	TM50747	30-Mar-17	*Mastomys natalensis s.l.*	Limpopo	F	Fe/ Ki	Neg	Neg
UP 12240	UPR 362	TM50748	30-Mar-17	*Saccostomus campestris*	Limpopo	M	Ki	Neg	Neg
UP 12241	UPR 363	Released; no voucher	30-Mar-17	*Steatomys sp.*	Limpopo	M	Fe	Neg	Neg
UP 12242	UPR 364	TM50749	30-Mar-17	*Aethomys ineptus s.l.*	Limpopo	F	Fe/ Ki	Neg	Neg
UP 12243	UPR 365	Released; no voucher	30-Mar-17	*Gerbilliscus leucogaster*	Limpopo	M	Fe	Neg	Neg
UP 12244	UPR 366	TM50750	30-Mar-17	*Saccostomus campestris*	Limpopo	F	Fe/ Ki	Neg	Neg

UP 12245	UPR 367	Released; no voucher	30-Mar-17	*Gerbilliscus leucogaster*	Limpopo	F	Fe	Neg	Neg
UP 12246	UPR 368	TM50545	30-Mar-17	*Gerbilliscus leucogaster*	Limpopo	M	Fe/ Ki	Neg	Neg
UP 12247	UPR 369	Released; no voucher	30-Mar-17	*Gerbilliscus leucogaster*	Limpopo	M	Fe	Neg	Neg
UP 12248	UPR 370	TM50751	30-Mar-17	*Saccostomus campestris*	Limpopo	M	Fe/ Ki	Neg	Neg
UP 12249	UPR 371	Released; no voucher	30-Mar-17	*Gerbilliscus leucogaster*	Limpopo	F	Fe	Neg	Neg
UP 12250	UPR 372	Released; no voucher	30-Mar-17	*Gerbilliscus leucogaster*	Limpopo	F	Fe	Neg	Neg
UP 12251	UPR 373	Released; no voucher	30-Mar-17	*Steatomys sp.*	Limpopo	M	Fe	Neg	Neg
UP 12252	UPR 374	Released; no voucher	30-Mar-17	*Mastomys natalensis s.l.*	Limpopo	M	Fe	Neg	Neg
UP 12253	UPR 375	Released; no voucher	31-Mar-17	*Steatomys sp.*	Limpopo	F	Fe	Neg	Neg
UP 12254	UPR 376	TM50752	31-Mar-17	*Steatomys sp.*	Limpopo	M	Ki	Neg	Neg
UP 12255	UPR 377	TM50753	31-Mar-17	*Mastomys natalensis s.l.*	Limpopo	F	Ki	Neg	Neg
UP 12256	UPR 378	TM50754	31-Mar-17	*Graphiurus murinus*	Limpopo	F	Ki	Neg	Neg
UP 12257	UPR 379	Released; no voucher	31-Mar-17	*Mastomys natalensis s.l.*	Limpopo	F	Fe	Neg	Neg
UP 12258	UPR 380	TM50755	31-Mar-17	*Steatomys sp.*	Limpopo	F	Fe/ Ki	Neg	Neg
UP 12259	UPR 381	TM50546	31-Mar-17	*Gerbilliscus leucogaster*	Limpopo	F	Fe/ Ki	Neg	Neg
UP 12260	UPR 382	TM50756	31-Mar-17	*Steatomys krebsii*	Limpopo	F	Fe/ Ki	Neg	Neg
UP 12261	UPR 383	Released; no voucher	31-Mar-17	*Mastomys natalensis s.l.*	Limpopo	M	Fe	Neg	Neg
UP 12262	UPR 384	Released; no voucher	31-Mar-17	*Mastomys natalensis s.l.*	Limpopo	M	Fe	Neg	Neg
UP 12264	UPR 386	Released; no voucher	31-Mar-17	*Saccostomus campestris*	Limpopo	M	Fe	Neg	Neg
UP 12265	UPR 387	TM50757	29-Mar-17	*Lemniscomys rosalia*	Limpopo	F	Ki	Neg	Neg
UP 12266	UPR 388	TM50758	29-Mar-17	*Steatomys sp.*	Limpopo	M	Ki	Neg	Neg
UP 12267	UPR 389	TM50759	29-Mar-17	*Steatomys sp.*	Limpopo	F	Ki	Neg	Neg
UP 12268	UPR 390	TM50760	29-Mar-17	*Steatomys sp.*	Limpopo	M	Ki	Neg	Neg
UP 12269	UPR 391	TM50761	29-Mar-17	*Steatomys sp.*	Limpopo	M	Ki	Neg	Neg
UP 12270	UPR 392	TM50762	30-Mar-17	*Steatomys sp.*	Limpopo	F	Ki	Neg	Neg
UP 12271	UPR 393	TM49274	30-Mar-17	*Suncus lixus*	Limpopo	F	Ki	Neg	Neg
UP 12272	UPR 394	TM50763	30-Mar-17	*Steatomys sp.*	Limpopo	F	Ki	Neg	Neg
UP 12273	UPR 395	TM50764	30-Mar-17	*Mastomys natalensis s.l.*	Limpopo	F	Ki	Neg	Neg
UP 12274	UPR 396	TM50765	30-Mar-17	*Steatomys sp.*	Limpopo	F	Ki	Neg	Neg
UP 12275	UPR 397	TM50766	16-May-17	*Mus (Nannomys) sp.*	Limpopo	M	Ki	Neg	Neg
UP 12276	UPR 398	TM50767	17-May-17	*Mastomys natalensis s.l.*	Limpopo	M	Ki	Neg	Neg
UP 12277	UPR 399	TM50768	17-May-17	*Steatomys sp.*	Limpopo	F	Ki	Neg	Neg
UP 12278	UPR 400	TM50769	16-May-17	*Mastomys natalensis s.l.*	Limpopo	M	Ki	Neg	Neg
UP 12279	UPR 401	TM50770	17-May-17	*Mastomys natalensis s.l.*	Limpopo	M	Ki	Neg	Neg
UP 12280	UPR 402	NHCPHE_MAM-4	17-May-17	*Mastomys natalensis s.l.*	Limpopo	M	Ki	Neg	Neg
UP 12281	UPR 403	TM49275	17-May-17	*Suncus lixus*	Limpopo	F	Fe/ Ki	Neg	Neg
UP 12282	UPR 404	NHCPHE_MAM-12	17-May-17	*Mastomys natalensis s.l.*	Limpopo	F	Fe/ Ki	Neg	Neg
UP 12283	UPR 405	TM50771	17-May-17	*Steatomys sp.*	Limpopo	M	Ki	Neg	Neg
UP 12284	UPR 406	TM49276	19-May-17	*Suncus lixus*	Limpopo	M	Ki	Neg	Neg

UP 12285	UPR 407	Released; no voucher	16-May-17	*Aethomys sp./Micaelamys sp.*	Limpopo	M	Fe	Neg	Neg
UP 12289	UPR 409	Released; no voucher	16-May-17	*Saccostomus campestris*	Limpopo	M	Fe	Neg	Neg
UP 12291	UPR 410	NHCPHE_MAM-1	16-May-17	*Micaelamys namaquensis*	Limpopo	M	Fe/ Ki	**Pos**	Neg
UP 12292	UPR 411	Released; no voucher	16-May-17	*Aethomys/Micaelamys sp.*	Limpopo	M	Fe	Neg	Neg
UP 12294	UPR 412	TM50772	16-May-17	*Aethomys ineptus s.l.*	Limpopo	F	Fe/ Ki	Neg	Neg
UP 12295	UPR 413	TM50773	16-May-17	*Aethomys ineptus s.l.*	Limpopo	M	Ki	Neg	Neg
UP 12296	UPR 414	NHCPHE_MAM-24	16-May-17	*Gerbilliscus leucogaster*	Limpopo	M	Fe/ Ki	Neg	Neg
UP 12297	UPR 415	NHCPHE_MAM-5	16-May-17	*Gerbilliscus leucogaster*	Limpopo	F	Fe/ Ki	Neg	Neg
UP 12298	UPR 416	TM50774	16-May-17	*Mastomys natalensis s.l.*	Limpopo	M	Ki	Neg	Neg
UP 12299	UPR 417	NHCPHE_MAM-6	16-May-17	*Mus (Nannomys) minutoides s.l.*	Limpopo	F	Ki	Neg	Neg
UP 12300	UPR 418	TM50775	16-May-17	*Mastomys natalensis s.l.*	Limpopo	F	Fe/ Ki	Neg	Neg
UP 12301	UPR 419	TM50776	16-May-17	*Mastomys natalensis s.l.*	Limpopo	M	Ki	Neg	Neg
UP 12302	UPR 420	Released; no voucher	16-May-17	*Aethomys sp.*	Limpopo	M	Fe	Neg	Neg
UP 12303	UPR 421	TM50547	16-May-17	*Gerbilliscus leucogaster*	Limpopo	F	Fe/ Ki	Neg	Neg
UP 12304	UPR 422	Released; no voucher	16-May-17	*Aethomys ineptus s.l.*	Limpopo	M	Fe	Neg	Neg
UP 12305	UPR 423	TM50777	16-May-17	*Lemniscomys rosalia*	Limpopo	F	Fe/ Ki	Neg	Neg
UP 12306	UPR 424	Released; no voucher	16-May-17	*Aethomys ineptus s.l.*	Limpopo	M	Fe	Neg	Neg
UP 12307	UPR 425	TM50548	16-May-17	*Gerbilliscus leucogaster*	Limpopo	F	Fe/ Ki	Neg	Neg
UP 12310	UPR 427	TM50778	17-May-17	*Saccostomus campestris*	Limpopo	M	Fe/ Ki	Neg	Neg
UP 12311	UPR 428	TM50779	17-May-17	*Mastomys natalensis s.l.*	Limpopo	F	Fe/ Ki	Neg	Neg
UP 12312	UPR 429	TM50780	17-May-17	*Steatomys sp.*	Limpopo	M	Ki	Neg	Neg
UP 12313	UPR 430	TM50781	17-May-17	*Mastomys natalensis s.l.*	Limpopo	M	Fe/ Ki	Neg	Neg
UP 12314	UPR 431	TM50782	17-May-17	*Gerbilliscus leucogaster*	Limpopo	F	Ki	Neg	Neg
UP 12315	UPR 432	TM50783	17-May-17	*Mastomys natalensis s.l.*	Limpopo	F	Fe/ Ki	Neg	Neg
UP 12316	UPR 433	TM50784	17-May-17	*Mastomys natalensis s.l.*	Limpopo	F	Fe/ Ki	Neg	Neg
UP 12317	UPR 434	TM50785	17-May-17	*Mastomys natalensis s.l.*	Limpopo	F	Ki	Neg	Neg
UP 12318	UPR 435	TM50786	17-May-17	*Gerbilliscus leucogaster*	Limpopo	M	Ki	Neg	Neg
UP 12319	UPR 436	TM50787	17-May-17	*Micaelamys namaquensis*	Limpopo	F	Fe/ Ki	Neg	Neg
UP 12320	UPR 437	TM50788	17-May-17	*Gerbilliscus leucogaster*	Limpopo	F	Fe/ Ki	Neg	Neg
UP 12321	UPR 438	TM50789	17-May-17	*Aethomys/Micaelamys sp.*	Limpopo	F	Ki	Neg	Neg
UP 12323	UPR 440	Released; no voucher	17-May-17	*Mastomys natalensis s.l.*	Limpopo	F	Fe	Neg	Neg
UP 12327	UPR 443	TM50790	17-May-17	*Gerbilliscus leucogaster*	Limpopo	F	Ki	Neg	Neg
UP 12328	UPR 444	TM50791	17-May-17	*Steatomys sp.*	Limpopo	F	Ki	Neg	Neg
UP 12329	UPR 445	TM50792	17-May-17	*Mastomys natalensis s.l.*	Limpopo	M	Ki	Neg	Neg
UP 12330	UPR 446	TM50793	17-May-17	*Mastomys natalensis s.l.*	Limpopo	F	Ki	Neg	Neg
UP 12331	UPR 447	Released; no voucher	17-May-17	*Mastomys natalensis s.l.*	Limpopo	M	Fe	Neg	Neg
UP 12332	UPR 448	TM50794	17-May-17	*Mastomys natalensis s.l.*	Limpopo	M	Fe/ Ki	Neg	Neg
UP 12337	UPR 451	NHCPHE_MAM-7	18-May-17	*Steatomys sp.*	Limpopo	M	Fe/ Ki	Neg	Neg
UP 12338	UPR 452	TM50795	18-May-17	*Mastomys natalensis s.l.*	Limpopo	M	Ki	Neg	Neg

UP 12339	UPR 453	TM50796	18-May-17	*Steatomys sp.*	Limpopo	M	Ki	Neg	Neg
UP 12340	UPR 454	TM50797	18-May-17	*Steatomys sp.*	Limpopo	F	Ki	Neg	Neg
UP 12341	UPR 455	TM50798	18-May-17	*Steatomys sp.*	Limpopo	M	Ki	Neg	Neg
UP 12342	UPR 456	TM50799	18-May-17	*Steatomys sp.*	Limpopo	M	Ki	Neg	Neg
UP 12343	UPR 457	TM50800	18-May-17	*Aethomys ineptus s.l.*	Limpopo	M	Ki	Neg	Neg
UP 12344	UPR 458	TM50801	18-May-17	*Mastomys natalensis s.l.*	Limpopo	F	Fe/ Ki	Neg	Neg
UP 12345	UPR 459	TM50802	18-May-17	*Mastomys natalensis s.l.*	Limpopo	F	Ki	Neg	Neg
UP 12346	UPR 460	TM50803	18-May-17	*Saccostomus campestris*	Limpopo	F	Fe/ Ki	Neg	Neg
UP 12347	UPR 461	TM50804	18-May-17	*Lemniscomys rosalia*	Limpopo	F	Ki	Neg	Neg
UP 12348	UPR 462	TM50805	18-May-17	*Mastomys natalensis s.l.*	Limpopo	F	Fe/ Ki	Neg	Neg
UP 12350	UPR 464	Released; no voucher	18-May-17	*Gerbilliscus leucogaster*	Limpopo	M	Fe	Neg	Neg
UP 12351	UPR 465	Released; no voucher	18-May-17	*Mastomys natalensis s.l.*	Limpopo	M	Fe	Neg	Neg
UP 12354	UPR 468	Released; no voucher	18-May-17	*Gerbilliscus leucogaster*	Limpopo	F	Fe	Neg	Neg
UP 12356	UPR 470	Released; no voucher	18-May-17	*Aethomys ineptus s.l.*	Limpopo	F	Fe	Neg	Neg
UP 12358	UPR 472	Released; no voucher	18-May-17	*Mastomys natalensis s.l.*	Limpopo	F	Fe	Neg	Neg
UP 12360	UPR 474	Released; no voucher	18-May-17	*Mastomys natalensis s.l.*	Limpopo	F	Fe	Neg	Neg
UP 12361	UPR 475	Released; no voucher	18-May-17	*Mastomys natalensis s.l.*	Limpopo	F	Fe	Neg	Neg
UP 12362	UPR 476	Released; no voucher	18-May-17	*Steatomys sp.*	Limpopo	M	Fe	Neg	Neg
UP 12363	UPR 477	Released; no voucher	18-May-17	*Steatomys sp.*	Limpopo	M	Fe	Neg	Neg
UP 12365	UPR 478	Released; no voucher	18-May-17	*Aethomys ineptus s.l.*	Limpopo	F	Fe	Neg	Neg
UP 12366	UPR 479	TM50806	18-May-17	*Mus (Nannomys) sp.*	Limpopo	F	Ki	Neg	Neg
UP 12367	UPR 480	TM50807	18-May-17	*Mastomys natalensis s.l.*	Limpopo	M	Ki	Neg	Neg
UP 12368	UPR 481	TM50808	19-May-17	*Gerbilliscus leucogaster*	Limpopo	M	Fe/ Ki	Neg	Neg
UP 12369	UPR 482	NHCPHE_MAM-8	19-May-17	*Steatomys sp.*	Limpopo	M	Ki	Neg	Neg
UP 12370	UPR 483	TM50809	19-May-17	*Steatomys sp.*	Limpopo	M	Ki	Neg	Neg
UP 12371	UPR 484	TM50810	19-May-17	*Mastomys natalensis s.l.*	Limpopo	M	Ki	Neg	Neg
UP 12373	UPR 486	Released; no voucher	18-May-17	*Gerbilliscus leucogaster*	Limpopo	M	Fe	Neg	Neg
UP 12376	UPR 489	TM50716	18-May-17	*Mastomys natalensis s.l.*	Limpopo	M	Fe/ Ki	Neg	Neg
UP 12377	UPR 490	NHCPHE_MAM-9	19-May-17	*Mus (Nannomys) minutoides s.l.*	Limpopo	M	Fe/ Ki	Neg	Neg
UP 12378	UPR 491	TM50811	18-May-17	*Steatomys sp.*	Limpopo	F	Fe/ Ki	Neg	Neg
UP 12379	UPR 492	Released; no voucher	19-May-17	*Mastomys natalensis s.l.*	Limpopo	M	Fe	Neg	Neg
UP 12380	UPR 493	NHCPHE_MAM-10	19-May-17	*Aethomys ineptus*	Limpopo	M	Ki	Neg	Neg
UP 12381	UPR 494	TM50812	19-May-17	*Mastomys natalensis s.l.*	Limpopo	F	Fe/ Ki	Neg	Neg
UP 12382	UPR 495	Released; no voucher	19-May-17	*Mastomys natalensis s.l.*	Limpopo	M	Fe	Neg	Neg
UP 12384	UPR 497	Released; no voucher	19-May-17	*Mus (Nannomys) sp.*	Limpopo	M	Fe	Neg	Neg
UP 12384	UPR 500	TM50813	19-May-17	*Aethomys ineptus s.l.*	Limpopo	F	Ki	Neg	Neg
UP 12393	UPR 506	Released; no voucher	29-Aug-17	*Mastomys natalensis s.l.*	Limpopo	M	Fe	Neg	Neg

UP 12394	UPR 507	TM50814	29-Aug-17	*Mastomys natalensis s.l.*	Limpopo	M	Fe/ Ki	Neg	Neg
UP 12395	UPR 508	TM50815	29-Aug-17	*Mastomys natalensis s.l.*	Limpopo	M	Fe/ Ki	Neg	Neg
UP 12396	UPR 509	Released; no voucher	29-Aug-17	*Mastomys natalensis s.l.*	Limpopo	M	Fe	Neg	Neg
UP 12397	UPR 510	Released; no voucher	29-Aug-17	*Mastomys natalensis s.l.*	Limpopo	M	Fe	Neg	Neg
UP 12398	UPR 511	Released; no voucher	29-Aug-17	*Mus (Nannomys) sp.*	Limpopo	M	Fe	Neg	Neg
UP 12399	UPR 512	Released; no voucher	29-Aug-17	*Mastomys natalensis s.l.*	Limpopo	M	Fe	Neg	Neg
UP 12400	UPR 513	Released; no voucher	29-Aug-17	*Mastomys natalensis s.l.*	Limpopo	M	Fe	Neg	Neg
UP 12403	UPR 514	Released; no voucher	29-Aug-17	*Mastomys natalensis s.l.*	Limpopo	M	Fe	Neg	Neg
UP 12404	UPR 515	Released; no voucher	29-Aug-17	*Mastomys natalensis s.l.*	Limpopo	F	Fe	Neg	Neg
UP 12405	UPR 516	Released; no voucher	29-Aug-17	*Aethomys ineptus s.l.*	Limpopo	M	Fe	Neg	Neg
UP 12406	UPR 517	Released; no voucher	29-Aug-17	*Aethomys ineptus s.l.*	Limpopo	M	Fe	Neg	Neg
UP 12409	UPR 518	Released; no voucher	29-Aug-17	*Aethomys ineptus s.l.*	Limpopo	M	Fe	Neg	Neg
UP 12410	UPR 519	Released; no voucher	29-Aug-17	*Mastomys natalensis s.l.*	Limpopo	M	Fe	Neg	Neg
UP 12414	UPR 521	TM50816	29-Aug-17	*Elephantulus brachyrhynchus*	Limpopo	F	Fe/ Ki	Neg	Neg
UP 12415	UPR 522	TM50817	29-Aug-17	*Mastomys natalensis s.l.*	Limpopo	F	Ki	Neg	Neg
UP 12416	UPR 523	TM50818	29-Aug-17	*Mastomys natalensis s.l.*	Limpopo	F	Ki	Neg	Neg
UP 12417	UPR 524	TM50819	29-Aug-17	*Mastomys natalensis s.l.*	Limpopo	M	Fe/ Ki	Neg	Neg
UP 12418	UPR 525	NHCPHE_MAM-13	29-Aug-17	*Mastomys natalensis s.l.*	Limpopo	F	Fe/ Ki	Neg	Neg
UP 12419	UPR 526	TM50820	29-Aug-17	*Mus (Nannomys) sp.*	Limpopo	M	Ki	Neg	Neg
UP 12420	UPR 527	TM50821	29-Aug-17	*Mastomys natalensis s.l.*	Limpopo	M	Fe/ Ki	Neg	Neg
UP 12421	UPR 528	TM50822	29-Aug-17	*Mus (Nannomys) sp.*	Limpopo	F	Ki	Neg	Neg
UP 12422	UPR 529	Released; no voucher	29-Aug-17	*Aethomys ineptus s.l.*	Limpopo	M	Fe	Neg	Neg
UP 12423	UPR 530	Released; no voucher	29-Aug-17	*Aethomys sp.*	Limpopo	M	Fe	Neg	Neg
UP 12425	UPR 531	Released; no voucher	29-Aug-17	*Mastomys natalensis s.l.*	Limpopo	M	Fe	Neg	Neg
UP 12426	UPR 532	Released; no voucher	29-Aug-17	*Gerbilliscus leucogaster*	Limpopo	M	Fe	Neg	Neg
UP 12428	UPR 533	NHCPHE_MAM-11	29-Aug-17	*Lemniscomys rosalia*	Limpopo	M	Fe/ Ki	Neg	Neg
UP 12429	UPR 534	Released; no voucher	29-Aug-17	*Mastomys natalensis s.l.*	Limpopo	M	Fe	Neg	Neg
UP 12430	UPR 535	Released; no voucher	29-Aug-17	*Gerbilliscus leucogaster*	Limpopo	M	Fe	Neg	Neg
UP 12434	UPR 537	TM50823	29-Aug-17	*Aethomys ineptus s.l.*	Limpopo	F	Fe/ Ki	Neg	Neg
UP 12435	UPR 538	NHCPHE_MAM-15	29-Aug-17	*Aethomys ineptus*	Limpopo	M	Fe/ Ki	Neg	Neg
UP 12436	UPR 539	Released; no voucher	29-Aug-17	*Aethomys ineptus s.l.*	Limpopo	M	Fe	Neg	Neg
UP 12437	UPR 540	Released; no voucher	29-Aug-17	*Saccostomus campestris*	Limpopo	M	Fe	Neg	Neg
UP 12438	UPR 541	Released; no voucher	29-Aug-17	*Micaelamys namaquensis*	Limpopo	M	Fe	Neg	Neg
UP 12440	UPR 542	Released; no voucher	29-Aug-17	*Aethomys ineptus s.l.*	Limpopo	M	Fe	Neg	Neg

UP 12442	UPR 543	TM50824	29-Aug-17	*Aethomys ineptus s.l.*	Limpopo	M	Fe/ Ki	Neg	Neg
UP 12443	UPR 544	TM50825	29-Aug-17	*Lemniscomys rosalia*	Limpopo	F	Fe/ Ki	Neg	Neg
UP 12444	UPR 545	TM50826	30-Aug-17	*Mastomys natalensis s.l.*	Limpopo	M	Ki	Neg	Neg
UP 12445	UPR 546	TM50827	30-Aug-17	*Mastomys natalensis s.l.*	Limpopo	F	Ki	Neg	Neg
UP 12446	UPR 547	Released; no voucher	30-Aug-17	*Gerbilliscus leucogaster*	Limpopo	M	Fe	Neg	Neg
UP 12447	UPR 548	TM50828	30-Aug-17	*Micaelamys namaquensis*	Limpopo	F	Ki	Neg	Neg
UP 12448	UPR 549	Released; no voucher	30-Aug-17	*Gerbilliscus leucogaster*	Limpopo	F	Fe	Neg	Neg
UP 12449	UPR 550	Released; no voucher	30-Aug-17	*Aethomys ineptus s.l.*	Limpopo	F	Fe	Neg	Neg
UP 12450	UPR 551	Released; no voucher	30-Aug-17	*Mastomys natalensis s.l.*	Limpopo	M	Fe	Neg	Neg
UP 12452	UPR 552	Released; no voucher	30-Aug-17	*Aethomys ineptus s.l.*	Limpopo	M	Fe	Neg	Neg
UP 12453	UPR 553	Released; no voucher	30-Aug-17	*Aethomys ineptus s.l.*	Limpopo	M	Fe/ Ki	Neg	Neg
UP 12454	UPR 554	Released; no voucher	30-Aug-17	*Mus (Nannomys) sp.*	Limpopo	-	Fe	Neg	Neg
UP 12455	UPR 555	Released; no voucher	30-Aug-17	*Mus (Nannomys) sp.*	Limpopo	F	Fe	Neg	Neg
UP 12456	UPR 556	Released; no voucher	30-Aug-17	*Gerbilliscus leucogaster*	Limpopo	M	Fe	Neg	Neg
UP 12458	UPR 557	TM50829	30-Aug-17	*Mastomys natalensis s.l.*	Limpopo	F	Fe/ Ki	Neg	Neg
UP 12459	UPR 558	Released; no voucher	30-Aug-17	*Mastomys natalensis s.l.*	Limpopo	M	Fe	Neg	Neg
UP 12460	UPR 559	Released; no voucher	30-Aug-17	*Mastomys natalensis s.l.*	Limpopo	M	Fe	Neg	Neg
UP 12461	UPR 560	Released; no voucher	30-Aug-17	*Mus (Nannomys) sp.*	Limpopo	F	Fe	Neg	Neg
UP 12462	UPR 561	Released; no voucher	30-Aug-17	*Mus (Nannomys) sp.*	Limpopo	F	Fe	Neg	Neg
UP 12463	UPR 562	Released; no voucher	30-Aug-17	*Mus (Nannomys) sp.*	Limpopo	M	Fe	Neg	Neg
UP 12464	UPR 563	Released; no voucher	30-Aug-17	*Mastomys natalensis s.l.*	Limpopo	M	Fe	Neg	Neg
UP 12467	UPR 565	Released; no voucher	30-Aug-17	*Micaelamys namaquensis*	Meletse	M	Fe	Neg	Neg
UP 12468	UPR 566	Released; no voucher	30-Aug-17	*Aethomys ineptus s.l.*	Meletse	M	Fe	Neg	Neg
UP 12470	UPR 568	TM50830	31-Aug-17	*Mus (Nannomys) sp.*	Limpopo	F	Ki	Neg	Neg
UP 12471	UPR 569	TM50831	31-Aug-17	*Mastomys natalensis s.l.*	Limpopo	M	Ki	Neg	Neg
UP 12472	UPR 570	NHCPHE_MAM-2	31-Aug-17	*Mus (Nannomys) sp..*	Limpopo	F	Fe/ Ki	**Pos**	Neg
UP 12473	UPR 571	TM50832	31-Aug-17	*Mus (Nannomys) sp.*	Limpopo	F	Ki	Neg	Neg
UP 12474	UPR 572	TM50833	31-Aug-17	*Aethomys ineptus s.l.*	Limpopo	F	Fe/ Ki	Neg	Neg
UP 12475	UPR 573	TM50834	31-Aug-17	*Lemniscomys rosalia*	Limpopo	F	Fe/ Ki	Neg	Neg
UP 12476	UPR 574	Released; no voucher	31-Aug-17	*Mastomys natalensis s.l.*	Limpopo	M	Fe	Neg	Neg
UP 12477	UPR 575	TM50835	31-Aug-17	*Aethomys ineptus s.l.*	Limpopo	F	Fe/ Ki	Neg	Neg
UP 12478	UPR 576	Released; no voucher	31-Aug-17	*Mus (Nannomys) sp.*	Limpopo	M	Fe	Neg	Neg
UP 12479	UPR 577	Released; no voucher	31-Aug-17	*Mus (Nannomys) sp.*	Limpopo	F	Fe	Neg	Neg
UP 12480	UPR 578	Released; no voucher	31-Aug-17	*Mastomys natalensis s.l.*	Limpopo	M	Fe	Neg	Neg
UP 12481	UPR 579	Released; no voucher	31-Aug-17	*Mastomys natalensis s.l.*	Limpopo	M	Fe	Neg	Neg

UP 12482	UPR 580	Released; no voucher	31-Aug-17	*Mastomys natalensis s.l.*	Limpopo	M	Fe	Neg	Neg
UP 12483	UPR 581	Released; no voucher	31-Aug-17	*Mus (Nannomys) sp.*	Limpopo	F	Fe	Neg	Neg
UP 12484	UPR 582	Released; no voucher	31-Aug-17	*Gerbilliscus leucogaster*	Limpopo	M	Fe	Neg	Neg
UP 12485	UPR 583	Released; no voucher	31-Aug-17	*Mus (Nannomys) sp.*	Limpopo	M	Fe	Neg	Neg
UP 12486	UPR 584	Released; no voucher	29-Aug-17	*Aethomys ineptus s.l.*	Limpopo	M	Fe	Neg	Neg
UP 12487	UPR 585	Released; no voucher	29-Aug-17	*Mus (Nannomys) sp.*	Limpopo	M	Fe	Neg	Neg
UP 12488	UPR 586	Released; no voucher	29-Aug-17	*Gerbilliscus leucogaster*	Limpopo	F	Fe	Neg	Neg
UP 12489	UPR 587	Released; no voucher	29-Aug-17	*Aethomys ineptus s.l.*	Limpopo	F	Fe	Neg	Neg
UP 12490	UPR 588	Released; no voucher	29-Aug-17	*Mus (Nannomys) sp.*	Limpopo	M	Fe	Neg	Neg
UP 12491	UPR 589	Released; no voucher	29-Aug-17	*Aethomys ineptus s.l.*	Limpopo	M	Fe	Neg	Neg
UP 12492	UPR 590	Released; no voucher	29-Aug-17	*Aethomys ineptus s.l.*	Limpopo	F	Fe	Neg	Neg
UP 12493	UPR 591	Released; no voucher	30-Aug-17	*Gerbilliscus leucogaster*	Limpopo	M	Fe	Neg	Neg
UP 12494	UPR 592	Released; no voucher	30-Aug-17	*Gerbilliscus leucogaster*	Limpopo	M	Fe	Neg	Neg
UP 12495	UPR 593	Released; no voucher	30-Aug-17	*Gerbilliscus leucogaster*	Limpopo	M	Fe	Neg	Neg
UP 12496	UPR 594	Released; no voucher	30-Aug-17	*Gerbilliscus leucogaster*	Limpopo	M	Fe	Neg	Neg
UP 12497	UPR 595	Released; no voucher	30-Aug-17	*Aethomys ineptus s.l.*	Limpopo	M	Fe	Neg	Neg
UP 12498	UPR 596	Released; no voucher	30-Aug-17	*Gerbilliscus leucogaster*	Limpopo	F	Fe	Neg	Neg
UP 12499	UPR 597	Released; no voucher	30-Aug-17	*Gerbilliscus leucogaster*	Limpopo	F	Fe	Neg	Neg
UP 12500	UPR 598	Released; no voucher	30-Aug-17	*Gerbilliscus leucogaster*	Limpopo	M	Fe	Neg	Neg
UP 12501	UPR 599	Released; no voucher	30-Aug-17	*Aethomys ineptus s.l.*	Limpopo	F	Fe	Neg	Neg
UP 12502	UPR 600	Released; no voucher	30-Aug-17	*Gerbilliscus leucogaster*	Limpopo	F	Fe	Neg	Neg
UP 12503	UPR 601	Released; no voucher	31-Aug-17	*Aethomys ineptus s.l.*	Limpopo	M	Fe	Neg	Neg
UP 12504	UPR 602	Released; no voucher	31-Aug-17	*Gerbilliscus leucogaster*	Limpopo	M	Fe	Neg	Neg
UP 12505	UPR 603	Released; no voucher	31-Aug-17	*Aethomys ineptus s.l.*	Limpopo	M	Fe	Neg	Neg
UP 12507	UPR 605	Released; no voucher	31-Aug-17	*Aethomys ineptus s.l.*	Limpopo	M	Fe	Neg	Neg
UP 12508	UPR 606	Released; no voucher	31-Aug-17	*Gerbilliscus leucogaster*	Limpopo	F	Fe	Neg	Neg
UP 12510	UPR 608	Released; no voucher	31-Aug-17	*Gerbilliscus leucogaster.*	Limpopo	F	Fe	Neg	Neg
UP 12511	UPR 609	Released; no voucher	31-Aug-17	*Gerbilliscus leucogaster*	Limpopo	F	Fe	Neg	Neg
UP 12512	UPR 610	Released; no voucher	31-Aug-17	*Gerbilliscus leucogaster*	Limpopo	F	Fe	Neg	Neg
UP 12513	UPR 611	Released; no voucher	31-Aug-17	*Aethomys ineptus s.l.*	Limpopo	F	Fe	Neg	Neg
UP 12514	UPR 612	Released; no voucher	31-Aug-17	*Gerbilliscus leucogaster*	Limpopo	F	Fe	Neg	Neg

UP 12515	UPR 613	Released; no voucher	21-Nov-17	*Mastomys natalensis s.l.*	Limpopo	F	Fe	Neg	Neg
UP 12516	UPR 614	TM50836	21-Nov-17	*Lemniscomys rosalia*	Limpopo	F	Fe/ Ki	Neg	Neg
UP 12517	UPR 615	NHCPHE_MAM-25	21-Nov-17	*Gerbilliscus leucogaster*	Limpopo	M	Fe/ Ki	Neg	Neg
UP 12518	UPR 616	NHCPHE_MAM-26	21-Nov-17	*Gerbilliscus leucogaster*	Limpopo	M	Ki	Neg	Neg
UP 12519	UPR 617	TM50837	21-Nov-17	*Mastomys natalensis s.l.*	Limpopo	F	Ki	Neg	Neg
UP 12520	UPR 618	TM50838	21-Nov-17	*Lemniscomys rosalia*	Limpopo	M	Fe/ Ki	Neg	Neg
UP 12521	UPR 619	Released; no voucher	21-Nov-17	*Mastomys natalensis s.l.*	Limpopo	M	Fe	Neg	Neg
UP 12522	UPR 620	Released; no voucher	21-Nov-17	*Aethomys ineptus s.l.*	Limpopo	F	Fe	Neg	Neg
UP 12523	UPR 621	Released; no voucher	21-Nov-17	*Gerbilliscus leucogaster*	Limpopo	F	Fe	Neg	Neg
UP 12524	UPR 622	TM50549	21-Nov-17	*Gerbilliscus leucogaster*	Limpopo	M	Fe/ Ki	Neg	Neg
UP 12525	UPR 623	Released; no voucher	21-Nov-17	*Mastomys natalensis s.l.*	Limpopo	F	Fe	Neg	Neg
UP 12526	UPR 624	TM50550	21-Nov-17	*Gerbilliscus leucogaster*	Limpopo	M	Fe	Neg	Neg
UP 12527	UPR 625	Released; no voucher	21-Nov-17	*Gerbilliscus leucogaster*	Limpopo	M	Fe	Neg	Neg
UP 12528	UPR 626	TM50839	21-Nov-17	*Mus (Nannomys) sp.*	Limpopo	M	Fe/ Ki	Neg	Neg
UP 12529	UPR 627	TM50840	21-Nov-17	*Mus (Nannomys) sp.*	Limpopo	M	Ki	Neg	Neg
UP 12530	UPR 628	TM50841	21-Nov-17	*Mus (Nannomys) sp.*	Limpopo	F	Ki	Neg	Neg
UP 12531	UPR 629	TM50842	21-Nov-17	*Mus (Nannomys) sp.*	Limpopo	M	Fe/ Ki	Neg	Neg
UP 12532	UPR 630	TM50843	21-Nov-17	*Mus (Nannomys) sp.*	Limpopo	F	Ki	Neg	Neg
UP 12534	UPR 632	TM50844	22-Nov-17	*Mus (Nannomys) sp.*	Limpopo	M	Ki	Neg	Neg
UP 12535	UPR 633	TM50845	22-Nov-17	*Mus (Nannomys) sp.*	Limpopo	-	Ki	Neg	Neg
UP 12536	UPR 634	TM50846	22-Nov-17	*Mastomys natalensis s.l.*	Limpopo	F	Fe/ Ki	Neg	Neg
UP 12537	UPR 635	Released; no voucher	22-Nov-17	*Mus (Nannomys) sp.*	Limpopo	M	Fe	Neg	Neg
UP 12538	UPR 636	TM50847	22-Nov-17	*Lemniscomys rosalia*	Limpopo	F	Fe/ Ki	Neg	Neg
UP 12539	UPR 637	Released; no voucher	22-Nov-17	*Gerbilliscus leucogaster*	Limpopo	M	Fe	Neg	Neg
UP 12540	UPR 638	Released; no voucher	22-Nov-17	*Mastomys natalensis s.l.*	Limpopo	M	Fe	Neg	Neg
UP 12541	UPR 639	Released; no voucher	22-Nov-17	*Gerbilliscus leucogaster*	Limpopo	M	Fe	Neg	Neg
UP 12542	UPR 640	Released; no voucher	22-Nov-17	*Aethomys ineptus s.l..*	Limpopo	F	Fe	Neg	Neg
UP 12543	UPR 641	Released; no voucher	22-Nov-17	*Gerbilliscus leucogaster*	Limpopo	M	Fe	Neg	Neg
UP 12544	UPR 642	Released; no voucher	22-Nov-17	*Gerbilliscus leucogaster*	Limpopo	M	Fe	Neg	Neg
UP 12545	UPR 643	TM50848	23-Nov-17	*Mus (Nannomys) sp.*	Limpopo	-	Ki	Neg	Neg
UP 12546	UPR 644	TM50849	23-Nov-17	*Mus (Nannomys) sp.*	Limpopo	F	Ki	Neg	Neg
UP 12547	UPR 645	TM49277	23-Nov-17	*Crocidura hirta*	Limpopo	F	Fe/ Ki	Neg	Neg
UP 12548	UPR 646	Released; no voucher	23-Nov-17	*Mus (Nannomys) sp.*	Limpopo	M	Fe	Neg	Neg
UP 12549	UPR 647	TM50850	23-Nov-17	*Mus (Nannomys) sp.*	Limpopo	F	Fe/ Ki	Neg	Neg
UP 12550	UPR 648	Released; no voucher	23-Nov-17	*Mastomys natalensis s.l.*	Limpopo	M	Fe	Neg	Neg
UP 12551	UPR 649	TM50851	23-Nov-17	*Mastomys natalensis s.l.*	Limpopo	M	Fe/ Ki	Neg	Neg
UP 12552	UPR 650	NHCPHE_MAM-14	23-Nov-17	*Aethomys ineptus*	Limpopo	M	Fe/ Ki	Neg	Neg

UP 12553	UPR 651	Released; no voucher	23-Nov-17	*Gerbilliscus leucogaster*	Limpopo	M	Fe	Neg	Neg
UP 12554	UPR 652	Released; no voucher	23-Nov-17	*Gerbilliscus leucogaster*	Limpopo	F	Fe	Neg	Neg
UP 12555	UPR 653	Released; no voucher	23-Nov-17	*Aethomys ineptus s.l.*	Limpopo	M	Fe	Neg	Neg
UP 12556	UPR 654	TM50852	23-Nov-17	*Aethomys/Micaelamys sp.*	Limpopo	F	Fe/ Ki	Neg	Neg
UP 12557	UPR 655	TM50853	23-Nov-17	*Mus (Nannomys) sp.*	Limpopo	F	Ki	Neg	Neg
UP 12558	UPR 656	TM50715	23-Nov-17	*Saccostomus campestris*	Limpopo	F	Fe/ Ki	Neg	Neg
UP 12559	UPR 657	TM50854	24-Nov-17	*Mus (Nannomys) sp.*	Limpopo	-	Ki	Neg	Neg
UP 12560	UPR 658	TM50855	24-Nov-17	*Mastomys natalensis s.l.*	Limpopo	M	Ki	Neg	Neg
UP 12561	UPR 659	Released; no voucher	24-Nov-17	*Aethomys ineptus s.l.*	Limpopo	M	Fe	Neg	Neg
UP 12562	UPR 660	TM50856	24-Nov-17	*Mus (Nannomys) sp.*	Limpopo	M	Fe/ Ki	Neg	Neg
UP 12563	UPR 661	TM50857	24-Nov-17	*Mastomys natalensis s.l.*	Limpopo	F	Fe/ Ki	Neg	Neg

Table S2 Primers used in this study:

Primer name	Sequence 5-3	Primer use	Reference
CytB For	CGA AGC TTG ATA TGA AAA CCA ATC GTT	Cytochrome B barcoding	Greenberg et al. (2012)
CytB rev	TGT AGT TRT CWG GGT CHT CTA	Cytochrome B barcoding	Greenberg et al. (2012)
LVL 3359 A-plus primer	AGAATTAGTGAAAGGGAGAGCAATTC	Arenavirus surveillance	Vieth et al. (2007)
LVL 3359 D-plus primer	AGAATCAGTGAAAGGGAAAGCAATTC	Arenavirus surveillance	Vieth et al. (2007)
LVL 3359 G-plus primer	AGAATTAGTGAAAGGGAGAGTAACTC	Arenavirus surveillance	Vieth et al. (2007)
LVL 3754 A-minus primer	CACATCATTGGTCCCCATTTACTATGATC	Arenavirus surveillance	Vieth et al. (2007)
LVL 3754 D-minus primer	CACATCATTGGTCCCCATTTACTGTGATC	Arenavirus surveillance	Vieth et al. (2007)
HAN-L-F1	ATGTAYGTIAGTGCWGATGC	Hantavirus surveillance	Klempa et al. (2006)
HAN-L-R1	AACATTSYYTTCCACATITC	Hantavirus surveillance	Klempa et al. (2006)
HAN-L-F2	TGCWGATGCIACIAARTGGTC	Hantavirus surveillance	Klempa et al. (2006)
HAN-L-R2	CRTCRTCWGARTGRTGDGCAA	Hantavirus surveillance	Klempa et al. (2006)
LV-SJ 1-plus	GCACCGGGGATCCTAGGCATTTTTGGTTGC	Characterization	Günther et al., (2000)
LV-SJ 3402-minus	CGCACAGTGGATCCTAGGC	Characterization	Günther et al., (2000)
LVL1-plus	CGCACCGAGGATCCTAGGCATT	Characterization	Günther et al., (2000)
LVL7279-minus	CGCACCGGGGATCCTAGGCAAT	Characterization	Günther et al., (2000)
S410_Rev1_PCRA (848)	CGGAGCTGTCGCTTAGAGTCC	Characterization validation	This study
S410_Rev2_PCRA (805)	CTCCTAGAGATGTAGATTGATTTG	Characterization validation	This study
LV-SJ 1-plus_modified PCRA	GCACCGGGGATCCTAGGCAT	Characterization validation	This study
S410_For1_PCRB (2160)	GAGCATTTGGATCAAGAGAAGTC	Characterization validation	This study
S410_For2_PCRB (2202)	GAAGCTGAGAGTAGGTTAAGCC	Characterization validation	This study
LV-SJ 3402-modified PCRB	CGCACAGTGGATCCTAGGC	Characterization validation	This study
L410_For1_PCRC (4661)	AGTTAATTGACAGACTGCAACAGCGG	Characterization validation	This study
L410_Rev_PCRC (6032)	CCCAGAGAGATACAAAGAAATGGC	Characterization validation	This study
L410_For2_PCRC (4741)	TCACAACACTCTTATTACTAGCC	Characterization validation	This study
L410_For1_PCRD (6774)	GGTATTATTGTAGCAAGTTCTACC	Characterization validation	This study
L410_For2_PCRD (6807)	GGGTAGAAGTGTGATGCCAATCG	Characterization validation	This study
LVL 7279-minus PCRD	CGCACCGGGGATCCTAGGCAAT	Characterization validation	Günther et al., (2000)
LVL1-plus PCRE	CGCACCGAGGATCCTAGGCATT	Characterization validation	Günther et al., (2000)
L410_Rev1_PCRE (409)	GAGTTTGTCTGGAAGTGGAAAC	Characterization validation	This study
L410_Rev2_PCRE (379)	GGGACATCTATCGCTCACTGTG	Characterization validation	This study
S570_For1_PCRF (3010)	CCGCTAGCTTGGGGTCTTTCCG	Characterization validation	This study
S570_For2_PCRF (2970)	TGAGTTTCTCCAGATCGGCTGC	Characterization validation	This study
LV-SJ 3402-modified PCRF	CGCACAGTGGATCCTAGGC	Characterization validation	This study
LV-SJ 1-plus_modified PCRG	GCACCGGGGATCCTAGGCAT	Characterization validation	This study
S570_Rev1_PCRG (558)	CCATTGAAATCACAGCTCACAGC	Characterization validation	This study
S570_Rev2_PCRG (517)	CCTCACTGATAGATGCAGGTGTG	Characterization validation	This study
L570_For1_PCRI (6657)	CAGTAGCTTGAATAGTGAATCACG	Characterization validation	This study
L570_For2_PCRI (6724)	GTACATCGACCATCCACTATGG	Characterization validation	This study

LVL 7279-minus PCRI	CGCACCGGGGATCCTAGGCAAT	Characterization validation	Günther et al., (2000)
LVL1-plus PCRJ	CGCACCGAGGATCCTAGGCATT	Characterization validation	Günther et al., (2000)
L570_Rev1_PCRJ (715)	GAGATGTAGATGTTGCTCTGACGC	Characterization validation	This study
L570_Rev2_PCRJ (685)	CACCCAGCTGAAGAAAGATATCC	Characterization validation	This study
